# Sex Differences in Blood Pressure and Cognitive Aging: National Alzheimer’s Coordinating Center (NACC_

**DOI:** 10.1093/geroni/igaf122.4008

**Published:** 2025-12-31

**Authors:** Carlos Araujo Menendez, Alonzo Mendoza, Carolina L Costa, Ariana Stickel

**Affiliations:** San Diego State University, La jolla, California, United States; San Diego State University, San Diego, California, United States; San Diego State University, San Diego, California, United States; San Diego State University, San Diego, California, United States

## Abstract

**Introduction:**

Elevated blood pressure (BP) is a known risk factor for cognitive decline that fluctuates over time, yet most studies rely on single or few BP measures and do not examine potential sex differences. Therefore, we investigated the associations between longitudinal BP and cognitive aging trajectories in Latino adults.

**Methods:**

Participants included 800 Latino adults without dementia from the NACC (2015–2022, 28 sites) with an average of 7-years of repeated BP assessments. Mixed-effects regression models evaluated the effects of time-varying systolic (SYS), diastolic (DIAS), and pulse pressure (PP) on trajectories of global cognition, executive functioning, episodic memory, and language. Models sequentially adjusted for demographics (age, education, language, heritage, cognitive status) and health variables (time-varying BMI, baseline hypertension).

**Results:**

A significant DIAS × Age × Sex interaction emerged for executive functioning (β = 0.41, SE = 0.18, p = 0.02). Among females, higher diastolic BP predicted steeper age-related decline (slope=-0.06, p = 0.01), whereas in males the effect was attenuated and slightly protective (slope = +0.02, p = 0.04). For global cognition, higher PP predicted greater decline across both sexes (β=-0.36, SE = 0.13, p = 0.01). No other significant effects were observed.

**Conclusion:**

Over 7-years of follow-up, diastolic BP had sex-specific associations with executive functioning trajectories, while PP broadly predicted global cognitive decline regardless of sex. These findings highlight that ongoing BP monitoring and consideration of sex-specific risks are essential for identifying Latino individuals who are at risk of cognitive decline.

